# Triclinic polymorph of 4-[4-(4-formyl­phen­oxy)but­oxy]benzaldehyde

**DOI:** 10.1107/S1600536812050994

**Published:** 2012-12-22

**Authors:** Tomislav Balić, Berislav Marković, Ivana Balić

**Affiliations:** a Department of Chemistry, J. J. Strossmayer University, Osijek, Franje Kuhača 20, HR-31000 Osijek, Croatia

## Abstract

The title compound, C_18_H_18_O_4_, is a triclinic polymorph of the previously reported monoclinic polymorph [Han & Zhen (2005 ▶). *Acta Cryst.* E**61**, o4358–o4359]. In the crystal of the triclinic polymorph, molecules are linked by two pairs of C—H⋯O hydrogen bonds, forming a two-dimensional network parallel to (102), and enclosing loops with graph set motifs of *R*_2_^2^(8) and *R*_2_^2^(6).

## Related literature

For the monoclinic polymorph, see: Han & Zhen (2005 ▶). For related structures and the synthesis of similar compounds, see: Balić *et al.* (2012 ▶); Ma & Cao (2011 ▶); Dehno Khalaji *et al.* (2011 ▶); Narasimha Moorthy *et al.* (2005 ▶); Ilhan *et al.* (2007 ▶). For graph-set analysis of hydrogen bonds, see Bernstein *et al.* (1995 ▶).
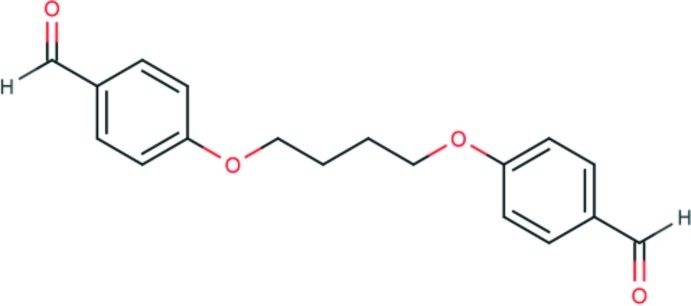


## Experimental

### 

#### Crystal data

C_18_H_18_O_4_
*M* *_r_* = 298.32
Triclinic, 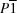

*a* = 4.4969 (2) Å
*b* = 7.9507 (6) Å
*c* = 11.0679 (8) Å
α = 73.854 (6)°
β = 84.788 (5)°
γ = 80.903 (5)°
*V* = 374.86 (4) Å^3^
*Z* = 1
Mo *K*α radiation
μ = 0.09 mm^−1^
*T* = 190 K
0.59 × 0.35 × 0.21 mm

#### Data collection

Oxford Diffraction Xcalibur Sapphire3 diffractometer
Absorption correction: multi-scan (*CrysAlis PRO*; Oxford Diffraction, 2009 ▶) *T*_min_ = 0.683, *T*_max_ = 1.000
2235 measured reflections
1473 independent reflections
1272 reflections with *I* > 2σ(*I*)
*R*_int_ = 0.010

#### Refinement

*R*[*F* ^2^ > 2σ(*F* ^2^)] = 0.043
*wR*(*F* ^2^) = 0.123
*S* = 1.04
1473 reflections
100 parameters
H-atom parameters constrained
Δρ_max_ = 0.29 e Å^−3^
Δρ_min_ = −0.17 e Å^−3^

### 

Data collection: *CrysAlis PRO* (Oxford Diffraction, 2009 ▶); cell refinement: *CrysAlis PRO*; data reduction: *CrysAlis PRO*; program(s) used to solve structure: *SIR2004* (Burla *et al.*, 2005 ▶); program(s) used to refine structure: *SHELXL97* (Sheldrick, 2008 ▶); molecular graphics: *ORTEP-3* (Farrugia, 2012 ▶); software used to prepare material for publication: *WinGX* (Farrugia, 2012 ▶), *PARST97* (Nardelli, 1995 ▶) and *Mercury* (Macrae *et al.*, 2006 ▶).

## Supplementary Material

Crystal structure: contains datablock(s) I, global. DOI: 10.1107/S1600536812050994/ng5308sup1.cif

Structure factors: contains datablock(s) I. DOI: 10.1107/S1600536812050994/ng5308Isup2.hkl

Supplementary material file. DOI: 10.1107/S1600536812050994/ng5308Isup3.cml

Additional supplementary materials:  crystallographic information; 3D view; checkCIF report

## Figures and Tables

**Table 1:** Hydrogen-bond geometry (Å, °)

*D*—H⋯*A*	*D*—H	H⋯*A*	*D*⋯*A*	*D*—H⋯*A*
C6—H6⋯O2^i^	0.95	2.58	3.4985 (16)	162
C1—H1⋯O1^ii^	0.95	2.59	3.3953 (18)	143

Symmetry codes: (i) 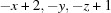; (ii) 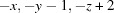.

**Fig. 1.**
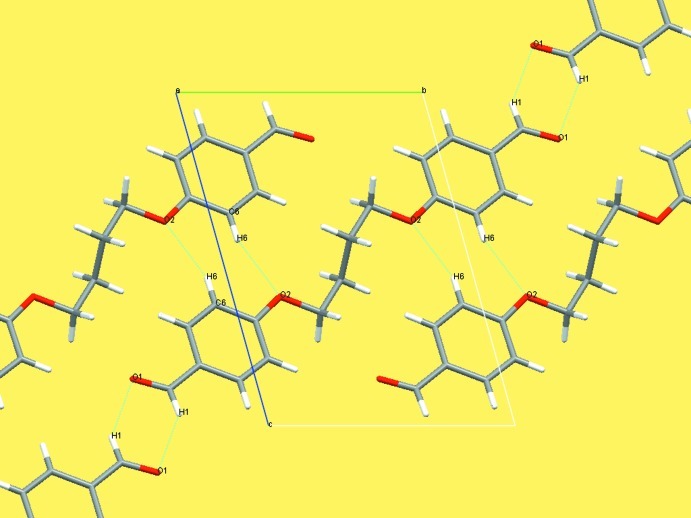
Crystal packing of title compound viewed down the a axis with dased lines representing weak C— H···O [graph set R22(6), R22(8)] intermolecular interactions.

**Fig. 2.**
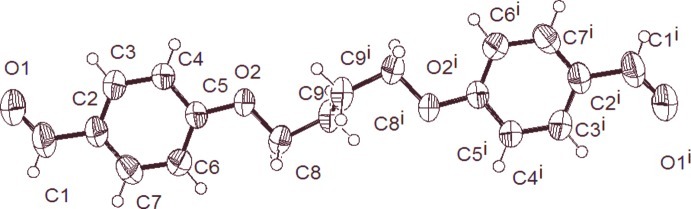
The molecular structure of the title compound with the atom numbering scheme. Displacement ellipsoids are drawn at the 50% probability level.

## supplementary crystallographic information

### Comment

Reacent structural studies of dialdehydes (Balić *et al.* 2012; Narasimha Moorthy *et al.* 2005), or the so called two-arm aldehydes have proposed them as potential precursors for condensation reactions with primary amines (Ilhan *et al.* 2007; Ma & Cao 2011; Dehno Khalaji *et al.* 2011). In a relation to this structural studies a new triclinic polymorph of title compound was found. Previously reported monoclinic polymorph (Han & Zhen 2005) was reported in *P*2_1_/*c* space group with *Z*=2. The new polymorph was found in *P*1 space group (*Z*=1), with different intermolecular interactions (Figure 1.).

The original polymorph crystallize in monoclinic space group *P*2_1_/c, with *a* = 7.988 (2), *b* = 6.6635 (16), *c*= 14.260 (4) Å, *β*= 96.354 (4)° and *Z* = 2 (Han & Zhen 2005). The title compound crystallizes in the space group *P*1 with *a* = 4.5749 (7), *b*= 7.9467 (10), *c* = 14.260 (4) Å, *α*= 73.597 (11)°, *β* = 83.154 (11)°, *γ* = 80.533 (12)° and *Z* = 1. In the reported structure crystallographic inversion centre lies in the center of the molecule, so the asymmetric unit comprises only one half of the molecule. The molecular structure of the title compound is shown in Figure 2. In the triclinic polymorph the molecules are linked in centrosymetric dimers *via* weak C1— H1···O1 intermolecular interactions, as previously reported by Narasimha Moorthy *et al.* (2005) and Balić *et al.* (2012). Additional stabilization of crystal structure is accomplished by weak C4— H4···O2 (Figure 1.). In the previously reported monoclinic polymorph the dihedral angle between benzaldehyde group and four central carbon atoms is 62.82°, while in triclinic polymorph this angle is 42.07°. However, the largest difference between these two polymorphs is manifested by the presence of *R^2^_2_*(6) and *R^2^_2_*(8) (Bernstein *et al.* 1995) supramolecular motifs in the triclinic polymorph.

### Experimental

The title compound was prepared by folowing procedure: *p*-hydroxybenzaldehyde (50 mmol) and K_2_CO_3_ (50 mmol) were mixed in DMF and the mixture was brought to brisk reflux. 25 mmol of butane-1,4-dibrom dissolved in DMF was then added and the reaction mixture was refluxed for 5 h. After the reaction was complete, 100 ml of water was added and the resulting percipitate was filtered and washed with water. Single crystals suitable for X-ray diffraction were grown *via* slow evaporation from ethanol solution of the title compound.

### Refinement

All H atoms, were positioned geometrically and refined using a riding model with C—H = 0.93 - 0.97 Å and with *U*_iso_(H) = 1.2 times *U*_eq_(C).

### Crystal data

**Table:**

C_18_H_18_O_4_	*Z* = 1
*M**_r_* = 298.32	*F*(000) = 158
Triclinic, *P*1	*D*_x_ = 1.321 Mg m^−^^3^
*a* = 4.4969 (2) Å	Mo *K*α radiation, λ = 0.7107 Å
*b* = 7.9507 (6) Å	Cell parameters from 1657 reflections
*c* = 11.0679 (8) Å	θ = 4.6–28.5°
α = 73.854 (6)°	µ = 0.09 mm^−^^1^
β = 84.788 (5)°	*T* = 190 K
γ = 80.903 (5)°	Block, colourless
*V* = 374.86 (4) Å^3^	0.59 × 0.35 × 0.21 mm

### Data collection

**Table:**

Oxford Diffraction Xcalibur Sapphire3 diffractometer	1473 independent reflections
Radiation source: Enhance (Mo) X-ray Source	1272 reflections with *I* > 2σ(*I*)
Graphite monochromator	*R*_int_ = 0.010
Detector resolution: 16.3426 pixels mm^-1^	θ_max_ = 26.0°, θ_min_ = 4.6°
ω scans	*h* = −5→4
Absorption correction: multi-scan (*CrysAlis PRO*; Oxford Diffraction, 2009)	*k* = −9→9
*T*_min_ = 0.683, *T*_max_ = 1.000	*l* = −13→13
2235 measured reflections	

### Refinement

**Table:**

Refinement on *F*^2^	Primary atom site location: structure-invariant direct methods
Least-squares matrix: full	Secondary atom site location: difference Fourier map
*R*[*F*^2^ > 2σ(*F*^2^)] = 0.043	Hydrogen site location: inferred from neighbouring sites
*wR*(*F*^2^) = 0.123	H-atom parameters constrained
*S* = 1.04	*w* = 1/[σ^2^(*F*_o_^2^) + (0.0676*P*)^2^ + 0.0807*P*] where *P* = (*F*_o_^2^ + 2*F*_c_^2^)/3
1473 reflections	(Δ/σ)_max_ < 0.001
100 parameters	Δρ_max_ = 0.29 e Å^−^^3^
0 restraints	Δρ_min_ = −0.17 e Å^−^^3^

### Special details

**Table:**

Experimental. (*CrysAlis PRO RED*; Oxford Diffraction, 2009)
Geometry. All e.s.d.'s (except the e.s.d. in the dihedral angle between two l.s. planes) are estimated using the full covariance matrix. The cell e.s.d.'s are taken into account individually in the estimation of e.s.d.'s in distances, angles and torsion angles; correlations between e.s.d.'s in cell parameters are only used when they are defined by crystal symmetry. An approximate (isotropic) treatment of cell e.s.d.'s is used for estimating e.s.d.'s involving l.s. planes.
Refinement. Refinement of *F*^2^ against ALL reflections. The weighted *R*-factor *wR* and goodness of fit *S* are based on *F*^2^, conventional *R*-factors *R* are based on *F*, with *F* set to zero for negative *F*^2^. The threshold expression of *F*^2^ > σ(*F*^2^) is used only for calculating *R*-factors(gt) *etc*. and is not relevant to the choice of reflections for refinement. *R*-factors based on *F*^2^ are statistically about twice as large as those based on *F*, and *R*- factors based on ALL data will be even larger.

### Fractional atomic coordinates and isotropic or equivalent isotropic displacement parameters (Å^2^)

**Table:**

	*x*	*y*	*z*	*U*_iso_*/*U*_eq_	
O1	0.2410 (3)	−0.49759 (14)	0.85962 (11)	0.0490 (4)	
O2	0.8584 (2)	0.19238 (11)	0.61242 (8)	0.0289 (3)	
C1	0.2367 (3)	−0.3608 (2)	0.88964 (14)	0.0377 (4)	
H1	0.1248	−0.3496	0.9647	0.045*	
C2	0.3920 (3)	−0.21240 (17)	0.81809 (13)	0.0283 (3)	
C3	0.3932 (3)	−0.06701 (18)	0.86379 (12)	0.0308 (3)	
H3	0.2870	−0.0627	0.9413	0.037*	
C4	0.5460 (3)	0.07238 (17)	0.79893 (12)	0.0280 (3)	
H4	0.5453	0.1711	0.8315	0.034*	
C5	0.7004 (3)	0.06505 (16)	0.68513 (12)	0.0242 (3)	
C6	0.6963 (3)	−0.07927 (18)	0.63690 (13)	0.0292 (3)	
H6	0.7991	−0.0831	0.5586	0.035*	
C7	0.5437 (3)	−0.21533 (17)	0.70273 (13)	0.0305 (3)	
H7	0.5410	−0.3130	0.6694	0.037*	
C8	0.8898 (3)	0.33927 (16)	0.65981 (12)	0.0268 (3)	
H8A	0.9914	0.2970	0.7406	0.032*	
H8B	0.6892	0.4045	0.6742	0.032*	
C9	1.0762 (3)	0.45809 (17)	0.56216 (12)	0.0276 (3)	
H9A	1.2708	0.3883	0.5457	0.033*	
H9B	1.1206	0.5532	0.5967	0.033*	

### Atomic displacement parameters (Å^2^)

**Table:**

	*U*^11^	*U*^22^	*U*^33^	*U*^12^	*U*^13^	*U*^23^
O1	0.0659 (8)	0.0366 (7)	0.0488 (7)	−0.0282 (5)	0.0138 (6)	−0.0119 (5)
O2	0.0378 (5)	0.0208 (5)	0.0297 (5)	−0.0128 (4)	0.0082 (4)	−0.0076 (4)
C1	0.0431 (8)	0.0373 (8)	0.0326 (8)	−0.0182 (6)	0.0064 (6)	−0.0048 (6)
C2	0.0292 (7)	0.0262 (7)	0.0282 (7)	−0.0087 (5)	−0.0004 (5)	−0.0026 (5)
C3	0.0335 (7)	0.0356 (8)	0.0233 (6)	−0.0095 (6)	0.0045 (5)	−0.0072 (6)
C4	0.0337 (7)	0.0255 (7)	0.0271 (7)	−0.0079 (5)	0.0007 (5)	−0.0094 (5)
C5	0.0245 (6)	0.0211 (6)	0.0257 (6)	−0.0057 (5)	0.0005 (5)	−0.0032 (5)
C6	0.0342 (7)	0.0254 (7)	0.0291 (7)	−0.0087 (5)	0.0073 (5)	−0.0095 (6)
C7	0.0352 (7)	0.0235 (7)	0.0346 (7)	−0.0101 (5)	0.0047 (6)	−0.0094 (6)
C8	0.0309 (7)	0.0215 (7)	0.0301 (7)	−0.0083 (5)	−0.0009 (5)	−0.0080 (5)
C9	0.0275 (6)	0.0230 (7)	0.0328 (7)	−0.0086 (5)	−0.0023 (5)	−0.0051 (6)

### Geometric parameters (Å, º)

**Table:**

O1—C1	1.2196 (18)	C5—C6	1.3969 (18)
O2—C5	1.3593 (15)	C6—C7	1.3704 (18)
O2—C8	1.4372 (15)	C6—H6	0.9500
C1—C2	1.4652 (18)	C7—H7	0.9500
C1—H1	0.9500	C8—C9	1.5105 (17)
C2—C3	1.3857 (19)	C8—H8A	0.9900
C2—C7	1.396 (2)	C8—H8B	0.9900
C3—C4	1.3871 (18)	C9—C9^i^	1.525 (3)
C3—H3	0.9500	C9—H9A	0.9900
C4—C5	1.3933 (18)	C9—H9B	0.9900
C4—H4	0.9500		
			
C5—O2—C8	118.23 (10)	C7—C6—H6	120.1
O1—C1—C2	124.62 (14)	C5—C6—H6	120.1
O1—C1—H1	117.7	C6—C7—C2	120.93 (13)
C2—C1—H1	117.7	C6—C7—H7	119.5
C3—C2—C7	118.64 (12)	C2—C7—H7	119.5
C3—C2—C1	120.77 (13)	O2—C8—C9	107.25 (10)
C7—C2—C1	120.58 (13)	O2—C8—H8A	110.3
C2—C3—C4	121.52 (12)	C9—C8—H8A	110.3
C2—C3—H3	119.2	O2—C8—H8B	110.3
C4—C3—H3	119.2	C9—C8—H8B	110.3
C3—C4—C5	118.82 (12)	H8A—C8—H8B	108.5
C3—C4—H4	120.6	C8—C9—C9^i^	113.78 (13)
C5—C4—H4	120.6	C8—C9—H9A	108.8
O2—C5—C4	124.74 (12)	C9^i^—C9—H9A	108.8
O2—C5—C6	115.04 (11)	C8—C9—H9B	108.8
C4—C5—C6	120.22 (12)	C9^i^—C9—H9B	108.8
C7—C6—C5	119.85 (12)	H9A—C9—H9B	107.7
			
O1—C1—C2—C3	−175.28 (14)	C3—C4—C5—C6	1.0 (2)
O1—C1—C2—C7	4.3 (2)	O2—C5—C6—C7	179.96 (11)
C7—C2—C3—C4	−1.3 (2)	C4—C5—C6—C7	−1.0 (2)
C1—C2—C3—C4	178.22 (12)	C5—C6—C7—C2	−0.2 (2)
C2—C3—C4—C5	0.2 (2)	C3—C2—C7—C6	1.4 (2)
C8—O2—C5—C4	5.11 (18)	C1—C2—C7—C6	−178.21 (12)
C8—O2—C5—C6	−175.90 (10)	C5—O2—C8—C9	179.31 (10)
C3—C4—C5—O2	179.95 (11)	O2—C8—C9—C9^i^	64.84 (16)

Symmetry code: (i) −*x*+2, −*y*+1, −*z*+1.

### Hydrogen-bond geometry (Å, º)

**Table:**

*D*—H···*A*	*D*—H	H···*A*	*D*···*A*	*D*—H···*A*
C6—H6···O2^ii^	0.95	2.58	3.4985 (16)	162
C1—H1···O1^iii^	0.95	2.59	3.3953 (18)	143

Symmetry codes: (ii) −*x*+2, −*y*, −*z*+1; (iii) −*x*, −*y*−1, −*z*+2.
